# Monitoring of the Single-Cell Behavior of an *Escherichia coli* Reporter Strain Producing L-phenylalanine in a Scale-Down Bioreactor by Automated Real-Time Flow Cytometry

**DOI:** 10.3390/biotech14030054

**Published:** 2025-07-03

**Authors:** Prasika Arulrajah, Sophi Katharina Riessner, Anna-Lena Heins, Dirk Weuster-Botz

**Affiliations:** 1 Chair of Biochemical Engineering, TUM School of Engineering and Design, Technical University of Munich, 85748 Garching, Germany; 2 Institute of Bioprocess and Biosystems Engineering, Hamburg University of Technology, 21073 Hamburg, Germany

**Keywords:** fed-batch, stirred-tank bioreactor, two-compartment bioreactor, automated real-time flow cytometry, *Escherichia coli* reporter strain, phenotypic population heterogeneity, residence time

## Abstract

Large-scale bioprocesses often suffer from spatial heterogeneities, which impact microbial performance and often lead to phenotypic population heterogeneity. To better understand these effects at the single-cell level, this study applied, for the first time, automated real-time flow cytometry (ART-FCM) to monitor L-phenylalanine production with an *Escherichia coli* triple reporter strain in a fed-batch process with glycerol as the carbon source. The strain was cultivated in both a well-mixed stirred-tank bioreactor (STR) and a scale-down two-compartment bioreactor (TCB), consisting of an STR and a coiled flow inverter (CFI) in bypass, to simulate spatial heterogeneities. ART-FCM enabled autonomous, high-frequency sampling every 20 min, allowing for real-time tracking of fluorescence signals linked to growth (*rrnB*-mEmerald), oxygen availability (*narGHIJ*-CyOFP1), and product formation (*aroFBL*-mCardinal2). The STR exhibited uniform reporter expression and higher biomass accumulation, while the TCB showed delayed product formation and pronounced phenotypic diversification depending on the set mean residence time in the CFI. Single-cell fluorescence distributions revealed that the shorter mean residence time in the CFI resulted in pronounced subpopulation formation, whereas longer exposure attenuated heterogeneity, indicating transcriptional adaptation. This finding highlights a critical aspect of scale-down studies: increased exposure duration to perturbations can enhance population robustness. Overall, this study demonstrates the relevance of ART-FCM, in combination with a multi-reporter strain, as a pioneering tool for capturing dynamic cellular behavior and correlating it to process performance, providing deeper insights into microbial heterogeneity under fluctuating bioprocess conditions.

## 1. Introduction

In the face of escalating climate change and environmental concerns, biotechnological production has emerged as a promising alternative to traditional chemical manufacturing processes, offering significant advantages in terms of sustainability and reduced greenhouse gas emissions [1,2]. However, the scale-up from laboratory scale to industrial scale production in bioreactors comes with challenges: In laboratory scale stirred-tank bioreactors (STRs), the culture medium is considered homogeneous, whereas with increasing operation scales (10 m^3^–800 m^3^), the quality of mixing deteriorates leading to gradients of process state variables in the bioreactor [3,4]. Consequently, cells experience different microenvironment dynamics travelling through the bioreactor to which they must adapt [5]. This can lead to the formation of a heterogeneous cell population out of an initial isogenic culture. Although phenotypic population heterogeneity is well-known in bioprocesses, especially in response to gradients or different kinds of stress, the mechanistic understanding of this phenomenon is poor [6].

To systematically investigate the cellular responses regarding these environmental fluctuations without the cost and complexity of large-scale production, scale-down bioreactors (SDBs) have been developed [7]. SDB are bioreactors designed to replicate the conditions and gradients (e.g., DO, pH or substrate) found in industrial scale bioprocesses. They can be designed as a single compartment or multi-compartment SDB, depending on the aim of the scale-down experiment. However, the main challenge remains in accurately simulating large-scale environmental gradients. The review article by Arulrajah et al. [8] provides a comprehensive analysis of different SDB setups with their distinct advantages and disadvantages.

One innovative approach for studying phenotypic population heterogeneity is using reporter strains, in which fluorescence proteins are coupled to expression of specific cellular characteristics [9]. This method allows the monitoring of individual cell properties by flow cytometry (FCM). In the recent study by Hoang et al. [10], a novel quadruple reporter strain was designed based on an engineered L-phenylalanine producing *E. coli* strain [11,12], which enabled simultaneous monitoring of the growth behavior, oxygen availability, L-phenylalanine formation, and general stress response of single-cells [10]. This *E. coli* strain was applied in a standardized fed-batch process [13] in an SDB consisting of an STR and a coiled flow inverter (CFI) in bypass to physically simulate the gradients occurring in large-scale bioreactors. However, the study relied on at-line FCM with manual sample preparation, limiting temporal resolution and excluding data collection during night hours. This leads to overseeing critical cellular responses at specific time points. One solution to overcome this limitation is automatizing the flow cytometry measurement.

Automated real-time flow cytometry (ART-FCM) has emerged as a powerful tool to address these limitations of traditional FCM in bioprocess monitoring. It enables continuous, operator-independent sampling and analysis, providing high-frequency data sets that capture cellular dynamics with the temporal resolution, which is potentially missed with traditional methods [14,15,16]. It was first introduced in the late 1990s by Zhao et al. (1999), who developed an automated flow injection flow cytometry to monitor the heterogeneity of protein expression in an *E. coli* cell population [17]. Since then, ART-FCM has been further developed by various research groups for individual applications in bioprocess monitoring and optimization [16]. For example, the research group of Frank Delvigne focuses on developing and applying ART-FCM techniques to study population dynamics and phenotypic diversification. Their newest technology is the so-called ’Segregostat’, which uses FCM data to control a microbial population’s degree of phenotypic diversification (e.g., for homogenizing the cell population for a given application) [18]. Another big field where ART-FCM is applied is continuously monitoring drinking water quality and wastewater treatment, offering real-time insights into microbial populations and enabling rapid detection of microbial contamination [19,20]. For this application field, the automation unit onCyt OC-300 (onCyt Microbiology AG; Dübendorf, Switzerland) was initially developed and used as an interface between different possible sample container and the flow cytometer, enabling automated sample collection at 15-min intervals, dying with specific fluorescence dyes, incubation at defined temperatures, and transferring the sample to the flow cytometer for fluorescence analysis [21]. This device was further developed and found in various applications in the biotechnology sector, including monitoring winemaking experiments, differentiating bacterial communities, and monitoring microalgal cocultures, to name some [22,23,24].

Despite these advancements, ART-FCM has not yet been applied in combination with a multi-reporter strain during a fed-batch cultivation in an SDB, where dynamic environmental fluctuations are simulated.

Our work presents, for the first time, the successful integration of ART-FCM with a triple reporter *E. coli* strain in a fed-batch bioprocess for L-phenylalanine production. The strain carries two chromosomally integrated fluorescent reporters, *rrnB*-mEmerald and *narGHIJ*-CyOFP1, and one plasmid-based reporter, *aroFBL*-mCardinal2. This enables monitoring of the growth behavior (*rrnB*-mEmerald), oxygen availability (*narGHIJ*-CyOFP1), and product formation (*aroFBL*-mCarindal2) [10]. Cultivation was performed in both a standardized fed-batch process in an STR, representing an ideally mixed and well-controlled compartment, and in a scale-down bioreactor, consisting of an STR and a CFI, to mimic the limitations and gradients occurring in industrial scale bioreactors. For the first time, ART-FCM was applied in both setups, where samples were taken autonomously every 20 min for FCM analysis to investigate phenotypic population heterogeneity under homogeneous and fluctuating environments. The aim of this study is to demonstrate the potential of combining ART-FCM with a multi-reporter strain to monitor single-cell dynamics in bioprocesses. This study specifically focused on how fluctuating environmental conditions impact phenotypic population heterogeneity and whether the exposure time to these limiting conditions affects adaptation behavior of the cells. It is hypothesized that cells exposed to dynamic conditions in the CFI will display increased heterogeneity compared to well-mixed conditions.

## 2. Materials and Methods

### 2.1. Strains and Cryopreservation

The quadruple reporter strain *E. coli* FUS4 (pF81_Kan_) 4RP (a modified strain of *E. coli* W3110 ∆*pheA*-*tyrA*-*aroF*, ∆*lacIZYA*::P_tac_-*aroFBL*; *rpoS*-mTagBFP2; *rrnB*-mEmerald; *narGHIJ*-CyOFP1 and *aroFBL*-mCardinal2) [10] was used in this study. However, due to the absence of the required excitation laser in the flow cytometer, the *rpos*-mTagBFP2 signal could not be detected. Therefore, the quadruple reporter strain is referred to as a triple reporter strain in this study. Cells were stored in a 20% glycerol stock at −80 °C.

### 2.2. Growth Medium and Preculture Preparation

Cryopreserved cells of the triple reporter strain were picked from the −80 °C freezer and immediately placed on a precooled cooling rank. A sterile inoculation loop was used to scratch frozen cell material, which was subsequently streaked on a minimal medium agar plate (5 g L^−1^ (NH_4_)_2_SO_4_, 3 g L^−1^ KH_2_PO_4_, 12 g L^−1^ K_2_HPO_4_, 0.1 g L^−1^ NaCl, 0.015 g L^−1^ CaCl_2_ · 2H_2_O, 0.1125 g L^−1^ (1.5 g L^−1^) iron (II) sulfate heptahydrate (buffered in tri-sodium citrate dihydrate), 0.3 g L^−1^ MgSO_4_ · 7 H_2_O and 0.0075 g L^−1^ Thiamine HCl, 0.075 g L^−1^ L-phenylalanine, 0.075 g L^−1^ L-tyrosine 0.05 g L^−1^ kanamycin, pH 7 with 3 M HCl, 20 g L^−1^ agar with 7 g L^−1^ glycerol as the carbon source prepared according to Weiner et al. [25]). The agar plate was sealed with parafilm and incubated at 37 °C for approximately 72 h. The two-step preculture preparation started with picking single colonies to inoculate three 100 mL flasks, each with 10 mL preculture minimal medium containing 7 g L^−1^ glycerol, and cultivated at 37 °C and 180 rpm in an orbital shaker (Multitron, Infors HT, Bottmingen, Switzerland) for approximately 30 h. The optical density at 600 nm (OD_600_) was measured with a spectrophotometer (Genesys 10 UV, Thermo Fisher Scientific, Waltham, MA, USA) to inoculate three 500 mL flasks containing 100 mL preculture minimal medium to a starting OD_600_ of 0.01. These flasks were again incubated at 37 °C at 250 rpm for around 24 h. The cells were harvested by centrifugation at 4 °C and 3260× *g* for 10 min (Rotixa 50 RS, Hettichlab, Tuttlingen, Germany). Afterward, the supernatant was discarded, and the pellet was resuspended in 5 mL fresh minimal medium without amino acids and glycerol. Bioreactor cultivations were inoculated to an initial OD_600_ of 0.05.

### 2.3. L-phenylalanine Production Process in the Stirred-Tank Bioreactor

The L-phenylalanine production process was first conducted in a fully controlled 3.6 L stirred-tank bioreactor (STR) (Labfors 5, Infors HT, Bottmingen, Switzerland), equipped with probes for regulating pH (EasyFerm Plus PHI Arc 325, Hamilton, Bonaduz, Switzerland) and dissolved oxygen (DO) (VisiFerm DO Arc 325 H0, Hamilton, Bonaduz, Switzerland). Furthermore, three baffles and three six-blade flat-blade turbines were mounted for optimal gas dispersion. Both probes were calibrated either with a two-point (pH 4.0 and pH 7.0) or with a one-point (100% DO) calibration before bioprocess start. Heat sterilization (121 °C, 20 min) was performed by filling deionized water (diH_2_O) in the STR. After sterilization, the cultivation medium, adapted from Weiner et al. [25], was aseptically transferred into the bioreactor, setting the initial cultivation volume to 1.35 L, the initial aeration rate to 1 vvm and the stirrer speed to 500 rpm. The temperature was regulated to 37 °C and pH 7.0 was kept constant by controlled addition of 25% ammonia or 42% phosphoric acid. Additionally, DO was kept above 30% air saturation by increasing the stirrer speed. After reaching the maximum speed of 1500 rpm, the aeration rate was increased in 0.1 L min^−1^ steps until the maximum of 5 L min^−1^.

The fed-batch process for L-phenylalanine production was divided into three phases: a batch phase, a two-step biomass production phase, and a product formation phase. The batch phase starts by inoculating the bioreactor to an OD_600_ of 0.05. After the complete depletion of the carbon source glycerol, the first phase of biomass production begins by applying an exponential feeding rate of the first feeding solution with a set growth rate of 0.1 h^−1^. The second biomass production phase starts exactly 9 h after the first feed was applied. Again, the second feeding solution was exponentially applied to keep the growth rate constant at 0.1 h^−1^. The subsequent product formation is initiated if DO cannot be held above 30% at maximum stirrer speed and aeration rate by inducing the cells with 0.3 mM IPTG. At the beginning of biomass production phases one and two, 6.75 and 13.5 mL of 4x concentrated minimal media were added to the cultivation media, respectively. This additive was a mixture of 20 g L^−1^ (NH_4_)_2_SO_4_, 12 g L^−1^ KH_2_PO_4_, 48 g L^−1^ K_2_HPO_4_, 0.4 g L^−1^ NaCl, 0.06 g L^−1^ CaCl_2_ · 2H_2_O, 0.45 g L^−1^ (4 g L^−1^) iron (II) sulfate heptahydrate (buffered in tri-sodium citrate dihydrate), 1.2 g L^−1^ MgSO_4_ · 7 H_2_O and 0.03 g L^−1^ Thiamine HCl. The first feed media consisted of 120 g L^−1^ Glycerol, 2.5 g L^−1^ L-phenylalanine, 3.6 g L^−1^ L-tyrosine and 60 g L^−1^ (NH_4_)_2_SO_4_ and 0.1 g L^−1^ kanamycin. The second, higher concentrated feed media contained 400 g L^−1^ glycerol, 1.11 g L^−1^ L-phenylalanine, 3.8 g L^−1^ L-tyrosine and 25 g L^−1^ (NH_4_)_2_SO_4_ and 0.1 g L^−1^ kanamycin. The first feed medium was titrated with 25% ammonia, and the second feed medium with 5 M KOH until the L-tyrosine was completely dissolved. The product formation phase was initiated by inducing the cells with 1 mL L^−1^ IPTG (0.3 M) together with 1 mL L^−1^ CaCl_2_ · 2H_2_O (15 g L^−1^), 1 mL L^−1^ MgSO_4_ · 7 H_2_O (300 g L^−1^), 1 mL L^−1^ Thiamine HCl (7.5 g L^−1^) and 5 mL L^−1^ iron (II) sulfate heptahydrate with tri-sodium citrate dihydrate (22.5 g L^−1^, 300 g L^−1^). The final feeding solution consisted of 800 g L^−1^ glycerol, 8 g L^−1^ (NH_4_)_2_SO_4,_ 8 g L^−1^ (NH_4_)_2_HPO_4_, and 16% (*v*/*v*) minimal medium without amino acids and glycerol. The omission of the amino acids L-phenylalanine and L-tyrosine in this feed medium was used to decouple growth from product synthesis, as the strain is auxotrophic for both amino acids. This limitation prevents further biomass formation and redirects the carbon flux towards L-phenylalanine production. A constant feeding rate of 0.18 glycerol g_CDW_^−1^ h^−1^ was applied. Samples for OD_600_, CDW, and HPLC were taken manually at defined process time points. Furthermore, every 20 min, samples were taken automatically from the bioreactor for automated real-time flow cytometry by the OC-300 automation add-on.

### 2.4. L-phenylalanine Production Process in the Two-Compartment Bioreactor

L-phenylalanine production in the two-compartment bioreactor (TCB) was generally conducted in the same way as in the STR. The construction was adapted from Hoang et al. [10]. The initial volume was maintained at 1.35 L, which was now split into 900 mL in the STR and 450 mL within the PFR. The STR was prepared as already described, with only two six-blade flat-blade turbines instead of three, having a 38 mm, and 78 mm distance to the bioreactor bottom, respectively. Furthermore, an additional dip tube was mounted on the vessel top plate for reflux from the CFI.

The STR was prepared and autoclaved, as already described in Section 2.3. To sterilize the CFI, the braided silicon tubes were put in an autoclavable box and vacuum sterilized (Laboratory autoclave LABOKLAV 160-MSLV, SHP Steriltechnik, Schloss Detzel, Germany). After sterilization, the silicon tubes were coiled around the frame, as described by Hoang et al. [10].

The process strategy for the production of L-phenylalanine was identical to the STR. The volumetric flow rates through the CFI bypass were set to 1.18 mL s^−1^, and 4.71 mL s^−1^, corresponding to a mean hydraulic residence time of 403 s, and 102 s, respectively, based on the working volume of the CFI [26]. These residence times were selected to cover the upper range of expected mixing times typically observed in large scale bioprocesses [27]. At a flow rate of 1.18 mL s^−1^, it would theoretically take approximately 19.1 min to pump the complete fermentation broth of 1.35 L through the CFI. When the flow rate is set to 4.71 mL s^−1^, it only takes around 4.8 min to do the same. OD_600_, CDW, HPLC, and ART-FC samples were taken from the STR.

### 2.5. OC-300 Automation Add-On for Automated Real-Time Flow Cytometry

For flow cytometry (FCM) analysis, a CytoFlex (Beckman Coulter, CA, USA) equipped with a 488 nm and a 638 nm laser was used. This device has nine detection filters for forward scatter (FSC), side scatter (SSC), and six bandpass filters for fluorescence detection (525/40 nm, 585/42 nm, 610/20 nm, 660/20 nm, and 2 × 780/60 nm). The fluorescence proteins mEmerald and CyOFP1 were excited by the 488 nm laser and detected at 525/40 and 585/42 nm, respectively. mCardinal2 was excited at 638 nm and detected at 660/20 nm. To eliminate background noise, the threshold was set on SSC-H and FSC-H with 10,000 and 7000, respectively.

**Figure 1:**
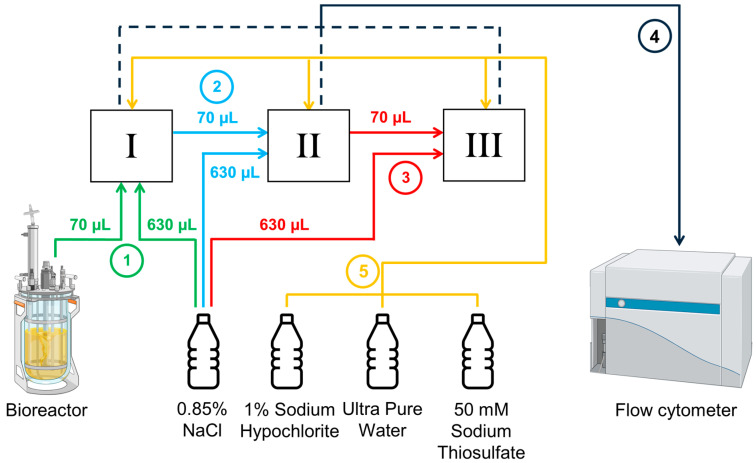
Schematic illustration of the sampling and dilution workflow for ART-FCM. The OC-300 Duo automation module is equipped with three dilution chambers (I, II, and III) for performing sequential serial dilutions. When dilution is required, 70 µL of biosuspension is drawn from the bioreactor (1) and mixed with 630 µL of 0.85% NaCl in chamber I. A 70 µL aliquot is then transferred to chamber II (2) and diluted again with 630 µL of 0.85% NaCl. For a 1:1000 dilution, the process is repeated in chamber III (3). For higher dilution factors, earlier chambers are emptied and reused for additional dilution steps. The final diluted sample is then transferred to the flow cytometer (4) for measurement. After each measurement cycle, all tubing and chambers are cleaned using designated cleaning solutions (5) to avoid cross-contamination. Created in BioRender. Heins, A. (2025) https://BioRender.com/x1uq4fs.

**Figure 2:**
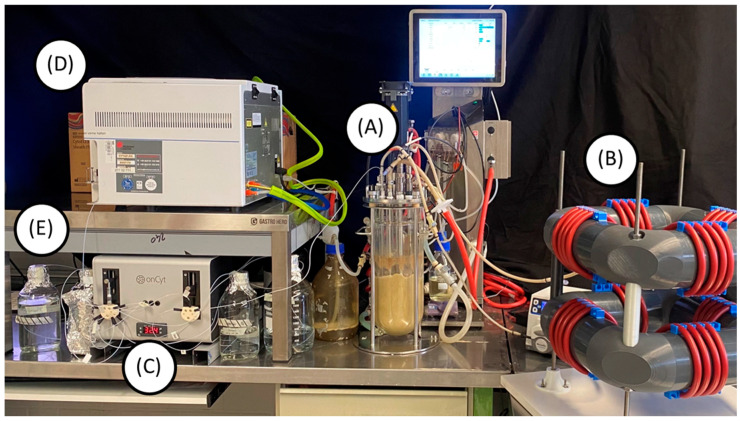
Photograph of the two-compartment bioreactor cultivation setup, consisting of a standard stirred-tank bioreactor (**A**) with a coiled flow inverter (**B**) in a bypass. The stirred-tank bioreactor is connected via a 120 mm long PTFE tubing to the right syringe pump of the onCyt^TM^ OC-300 automation add-on (**C**). The syringe takes samples from the bioreactor every 20 min. Depending on the cell concentration, the suspension is diluted with 0.85% NaCl in the chambers of the automation add-on (not visible). After dilution, the syringe pumps the sample into the flow cytometer (**D**) for measurement. After each measurement, all tubings are cleaned with 1% sodium hypochlorite, then with 50 mM sodium thiosulfate and finally rinsed with ultrapure water (**E**).

This setup was equipped for automated real-time flow cytometry (ART-FC) by integrating an OC-300 Duo automation module (onCyt Microbiology, Zürich, Switzerland). This device allowed the automation of flow cytometry analysis, sampling every 20 min, and tracking of fluorescence distributions. During online measurements, the OC-300 and the flow cytometer were controlled by the cyOn control software (Version V3.12.0) (onCyt Microbiology, Zürich, Switzerland). The system featured two valves. Valve one is connected to the FCM and valve two is connected to the STR. The connection to the STR was enabled via a 120 cm long hose with a diameter of 0.8 mm, which was inserted through a stainless-steel tube into the STR. The script used by the cyOn software specified that sampling should take place every 20 min. Before sampling, the dead volume from the sampling tube was entirely discarded (600 μL). Afterward, 700 μL of the sample is extracted from the bioreactor and transferred via the syringe to the flow cytometer. The measurement lasts precisely 67 s. If the sample exceeds the threshold count of 1000 events μL^−1^, dilution is applied in series. For the first 1:10 dilution, 70 μL sample is diluted with 630 μL 0.85% NaCl in one of the three chambers inside the OC-300. For the 1:100 dilution, first, a 1:10 dilution is prepared, and then 70 μL of that is mixed with 630 μL fresh NaCl in the second chamber. The 1:1000 is prepared the same way in the third chamber. For higher dilutions, the previously used chambers are emptied and used for further dilution steps. The OC-300 can perform dilutions up to 1:6000. To minimize sample carryover between each measurement, all tubes, chambers, and the syringe are cleaned automatically following the same protocol: first, all tubes and chambers are cleaned with 1% sodium hypochlorite, then with 50 mM sodium thiosulfate, and as a final step, rinsed with ultrapure water (Figure 1 and Figure 2).

### 2.6. Analysis of Optical Density and Biomass Concentration

The optical density was measured in triplicates using phosphate-buffered saline solution (PBS) as a reference at a wavelength of 600 nm using a spectrophotometer (Genesys 10 UV, Thermo Fisher Scientific, Waltham, MA, USA). The linear range of this photometer is between 0.05 and 0.3. Samples exceeding this range were diluted appropriately with PBS to ensure accuracy.

The cell dry weight (CDW) concentration was also estimated gravimetrically in triplicates. For that, 2 mL reaction tubes were dried at 80 °C for around 24 h, cooled to room temperature, weighed using a precision scale (Mettler Toledo, Excellence, XA204 DeltaRange^®^, Greifensee, Switzerland), and stored at room temperature until use. For CDW estimation, 2 mL of biosuspension was pipetted into the weighed tubes and centrifuged at 4 °C and 21,130× *g* for 20 min (Centrifuge 2424 R, Eppendorf, Hamburg, Germany). Supernatants were used for HPLC samples. Tubes with the remaining cell pellets were dried at 80 °C for at least 48 h and weighed again. The CDW concentration was calculated from the mass difference.

### 2.7. Quantification of Sugars, Organic Acids, and Amino Acids

For the quantification of sugars, organic acids, and amino acids by high-performance liquid chromatography (HPLC), the remaining supernatant of CDW estimation was used. The supernatant was filtered to remove the remaining cells through a 0.22 µm filter (Chromafil RC20/15 MS, Macherey-Nagel GmbH & Co.KG, Düren, Germany) and stored in 1.5 mL safe-lock reaction tubes (Eppendorf SE, Hamburg, Germany) at −20 °C until use.

To quantify the organic compounds, filtered supernatants were thawed and transferred into glass vials. The HPLC (LC-2030C Plus, Shimadzu Corp., Kyoto, Japan) was equipped with an ion exchange column (Aminex HPX-87 H, Bio-Rad Laboratories Inc., Hercules, CA, USA) as stationary phase and was kept constant at 60 °C. For the mobile phase, a filtered 5 mM H_2_SO_4_ solution was applied at an isocratic flow rate of 0.6 mL min^−1^ and 30 min, and the operating pressure was 43 bar. 10 µL of the sample was injected, and the elution of the distinct compounds was monitored by a refractive index detector (RID-20A, Shimadzu Corp., Kyoto, Japan). Standards with defined concentrations of the different compounds were prepared separately to calculate sample concentrations.

The central lab for chemical analysis at the Hamburg University of Technology carried out amino acid quantification. The method was based on OPA-derivatization and subsequent measurement with HPLC-FLD [28].

### 2.8. Evaluation of Flow Cytometry Data

The flow cytometry data produced with ART-FCM was saved continuously as .fcs data. Flow cytometry data were analyzed using FCS Express 7 (Version 7.24.0030) (De Novo Software, Pasadena, CA, USA), which includes calculating the mean and median values and generating all stacked-offset histogram plots. The fluorescence density plots were created by merging all .fcs files into one and plotted as density plots, where the x-axis indicates the processing time and the y-axis the fluorescence intensity plotted exponentially. To assess population heterogeneity, mean-to-median ratios (MMR) were calculated for each histogram. A ratio of 1 indicates a predominantly homogeneous population with a unimodal distribution, while increasing values suggest a greater degree of variability between individual cells.

## 3. Results

### 3.1. Comparison of Process Performances in STR and TCB

**Figure 3:**
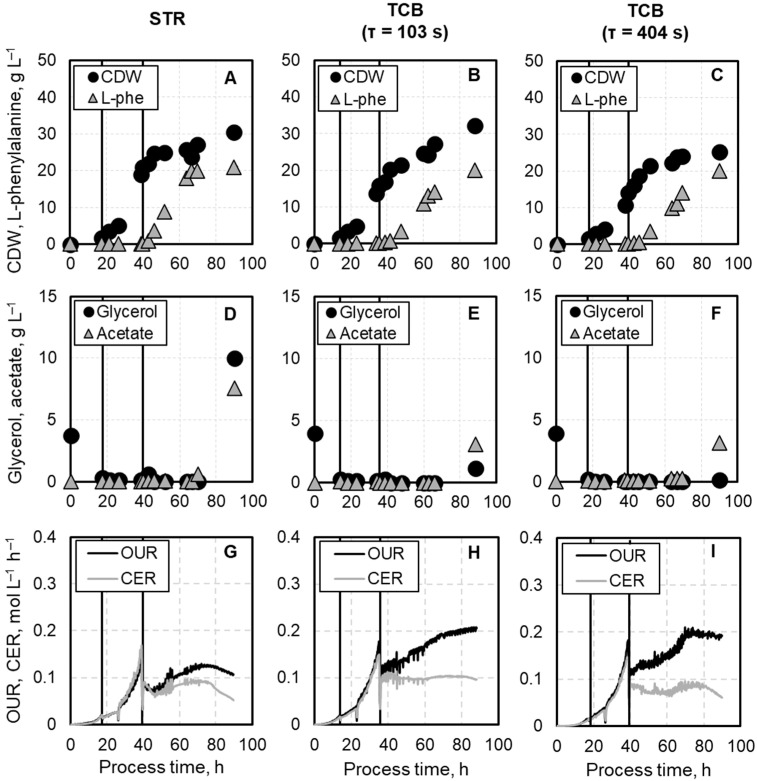
Comparison of the process performance of the L-phenylalanine production with *E. coli* FUS4 (pF81_Kan_) 3RP in a STR and TCB with mean residence times of 102 s, and 403 s in the CFI, respectively. (**A**–**C**) show the cell dry weight (CDW) together with the L-phenylalanine concentration (L-phe), while (**D**–**F**) depicts the substrate glycerol together with the byproduct acetate. (**G**–**I**) present the oxygen uptake rate (OUR) and carbon dioxide evolution rate (CER). The first vertical line indicated the end of the initial batch phase and the start of the biomass production phase. The second vertical depicts the start of the L-phenylalanine production phase.

The L-phenylalanine production process is divided into three phases: an initial batch phase, a two-step biomass production phase, and the product formation phase. The cultivation was conducted in a well-mixed stirred-tank bioreactor (STR) and in a two-compartment bioreactor (TCB) established by Hoang et al. [10]. In this scale-down bioreactor setup, the cultivation broth is continuously circulated between the homogeneous and well-controlled STR and the non-controlled zone in the coiled flow inverter, testing mean residence times of 102 s, and 403 s, respectively, within the CFI. The CFI was operated under laminar flow conditions, as indicated by Reynolds numbers of 1000 (for 102 s) and 250 (for 403 s). Effective radial mixing with low axial dispersion was confirmed by high Bodenstein numbers of 337 ± 12.81, and 164 ± 0.53, respectively [26]. This number indicates axial dispersion, with higher values representing narrower residence time distributions, even at laminar flow [26]. The production strain used in this study is auxotrophic for both L-phenylalanine and L-tyrosine, necessitating the supplementation of these amino acids during the batch and two-step biomass production phase to enable growth. The third feed medium, applied during the L-phenylalanine production phase, did not contain any amino acids, enabling growth-decoupled product synthesis. The initial batch phase lasted in all three cultivations between 14–18 h, reaching biomass concentrations of 1.73 ± 0.10 g L^−1^ in the STR, 1.55 ± 0.13 g L^−1^ in the TCB with the lowest mean residence time and 1.50 ± 0.26 g L^−1^ in the TCB with the highest mean residence time (Figure 3A–C).

**Figure A1:**
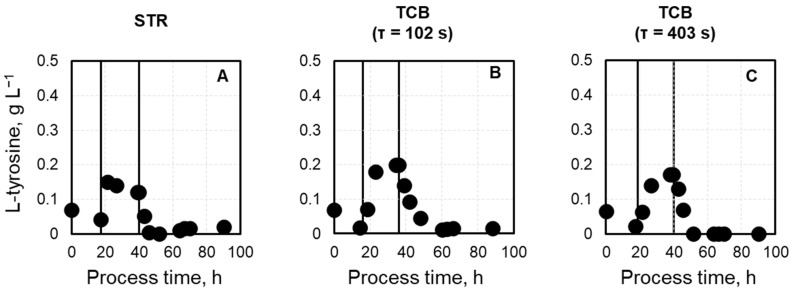
Concentration of L-tyrosine during the L-phenylalanine production process with *E. coli* FUS4 (pF81_Kan_) 3RP in an STR (**A**), a TCB with τ = 102 s in the CFI (**B**), and a TCB with τ = 403 s in the CFI (**C**). The first vertical line indicates the end of the batch and the start of the biomass production phase, while the second vertical line marks the start of the product formation phase.

Significant cell growth differences were observed with the initiation of the subsequent two-step biomass production phase. During the first exponential feeding phase, lasting in all cultivations for precisely nine hours, biomass concentrations showed first deviations with a trend of decreasing biomass concentration with increasing mean residence time in the CFI. In the STR, a biomass concentration of 5.03 ± 0.36 g L^−1^ was achieved, while in the TCB only 4.65 ± 0.40 g L^−1^ at a mean residence time of 102 s, and 4.10 ± 0.36 g L^−1^ at 403.30 s could be achieved. Major differences in biomass concentration were noticeable in the second phase of biomass production. This phase ended not after a fixed time but whenever the dissolved oxygen concentration inside the STR could not be kept above 40% air saturation at maximum stirrer speed and aeration rate. For the cultivation in the STR, this phase lasted 13.41 h, reaching a biomass concentration of 21.05 ± 0.93 g L^−1^. In contrast, in the TCB with the short mean residence time in the CFI (τ = 102 s), this phase was reduced to 12.75 h, reaching a biomass concentration of 15.88 ± 0.44 g L^−1^. For the cultivation with the longer mean residence time (τ = 403 s), this phase was similar in duration (12.92 h) but reached only a biomass concentration of 14.07 ± 1.00 g L^−1^. Although only the fed-batch cultivation in the STR reached the minimum biomass concentration of 20 g L^−1^ prior to the onset of oxygen limitation, the product formation was initiated in all processes by the addition of 0.3 mM IPTG. IPTG was added at the point when both the stirrer speed and aeration rate had reached their maximum setpoints. In the STR, this coincided with achieving the desired biomass concentration. In contrast, in the TCB cultivations, the desired biomass concentration was not achieved, yet induction was still performed. Due to the remaining L-tyrosine in the fermentation broth (Figure A1), the *E. coli* cells maintained to grow at the beginning of the L-phenylalanine production phase but remained at a relatively constant value afterward. The cultivation in the STR led to a maximum biomass concentration of 30.40 ± 0.53 g L^−1^. In comparison, the TCB cultivation with a mean residence time of 102 s led to a 7% higher, and the TCB cultivation with a residence time of 403.30 s led to a 20% lower maximum biomass concentration (Figure 3A–C).

The L-phenylalanine production in the STR cultivation started six hours after induction, and achieved the maximum product concentration of 21 g L^−1^ after 27 h. However, the fed-batch cultivations in the TCB showed longer adaptation times, resulting in the initiation of product formation about 12 h after induction. The maximum L-phenylalanine concentration of 20 g L^−1^ in both fed-batch processes in the TCB was only achieved at the end of the cultivation at a process time of around 90 h (Figure 3A–C). In all cultivations, the byproduct acetate and the carbon source glycerol began accumulating after achieving the maximum product concentration (Figure 3D–F).

The oxygen uptake rate (OUR) and the carbon dioxide evolution rate (CER) in the STR cultivation showed similar patterns. During the batch and biomass production phase, an exponential increase in both rates were observed. At the end of the biomass production phase, both rates reached their maximum of 0.15–0.17 mol L^−1^ h^−1^. With the induction of product formation, both rates dropped to lower values but increased afterward as long as L-phenylalanine was formed. Finally, a decrease in both rates took place. Similar patterns were observed in the fed-batch cultivations in the TCB, with the main difference being that the OUR has its maximum during the product formation phase with 0.21 mol L^−1^ h^−1^ for both TCB cultivations. In contrast, the curve for CER is comparable to the one from the STR process (Figure 3G–I).

### 3.2. Evaluation of the Median Fluorescence Intensities (MFI) and Correlation to Specific Process Features

**Figure 4:**
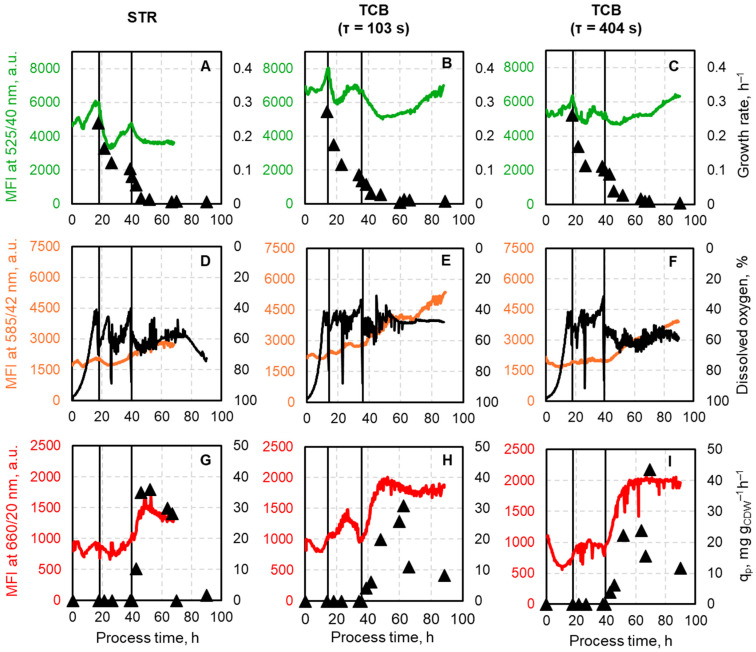
Real-time median fluorescence intensity (MFI) signals during the L-phenylalanine production process with *E. coli* FUS4 (pF81_Kan_) 3RP in an STR (first column), a TCB with τ = 102 s in the CFI (second column), and a TCB with τ = 403 s in the CFI (third column). (**A**–**C**) show the expression of the green fluorescing mEmerald, which is coupled to the *rrnB*-operon and the specific growth rate. (**D**–**F**) illustrates the signal of the orange fluorescing CyOFP1, which is coupled to the *narGHIJ*-operon and the dissolved oxygen level in the STR. The graphs (**G**–**I**) depict the red fluorescing mCardinal2 which is used to monitor the product formation by integrating this marker in the production plasmid together with the product formation rates. The first vertical line indicates the end of the batch and the start of the biomass production phase, while the second vertical line marks the start of the product formation phase.

**Monitoring growth behavior with green fluorescing mEmerald**. The green fluorescence mEmerald was used to follow the growth of the *E. coli* triple reporter strain by measuring its emission at 525/20 nm and plotted with the specific growth rate (Figure 4A–C). In general, all fed-batch processes showed the same pattern regarding the growth rate: the maximum growth rate of *E. coli* was achieved at the end of the batch phase, followed by a reduction to approximately 0.1 h^−1^ during the biomass production phase, and a further decline to near zero during the L-phenylalanine production phase. The trend for the mEmerald fluorescence was expected to follow the growth rate pattern. Indeed, the highest fluorescence intensity was detected at the end of the batch phase with median values of 6084.55, 8045.60, and 6360.30 for the cultivation in the STR, TCB (102 s), and TCB (403 s), respectively. During the first phase of biomass production, a rapid decline in fluorescence intensity was observed in all processes. Notably, with the onset of the second biomass production phase, an increase in fluorescence intensity became obvious in all cultivations, reaching values of 4760.75 (STR), 6708.80 (TCB with τ = 102 s), and 5360.75 (TCB with τ = 403 s). During the first 30 h of the product formation phase, mEmerald fluorescence followed the growth rate trend in all bioprocesses. Afterward, for the cultivations in the TCB, an increase in fluorescence intensity was detected, although the growth rate stayed near zero, and reached comparable high intensities at the end of the L-phenylalanine production processes as at the end of the batch phase.

**Monitoring oxygen availability with orange fluorescing CyOFP1.** The orange fluorescing CyOFP1 expression, detected at 585/42 nm, indicates oxygen availability and should, therefore, correlate with the dissolved oxygen (DO) concentration (Figure 4D–F). The DO concentration inside the STR was controlled to be held above 30% air saturation, whereas the DO concentration inside the CFI was neither controlled nor measured. The orange fluorescing reporter protein is expressed whenever cells experience low oxygen concentration. Therefore, the axis for the online measured dissolved oxygen concentration was plotted reversely to identify whether there is a correlation between fluorescence expression and the DO trend. During batch and mid biomass production phase, the median fluorescence intensities of CyOFP1 in the STR showed minor fluctuations and stayed relatively constant at a median value of around 1858.08 ± 104.51. Towards the end of biomass production phase, where the DO concentration approached a value of 40% air saturation, a rise in fluorescence intensity can be seen. Upon IPTG induction, median fluorescence increased further, reaching a maximum value of 2934 and showing a trend to lower intensities afterward. Both TCB cultivations show similar trends with minor changes during batch and biomass production phases. In comparison to the reference fed-batch process in the STR, the induction of L-phenylalanine production leads to a steeper increase in median fluorescence intensity. After a process time of 70 h in both fed-batch production processes, a stagnation of CyOFP1 fluorescence was observed first, followed by a rapid increase toward process termination.

**Monitoring of product formation with red fluorescing mCardinal2.** Product formation can be followed by the expression of the plasmid-based marker protein mCardinal2, detected at 660/20, shown together with the biomass specific L-phenylalanine formation rate (Figure 4G–I). In all cultivations, an expression of this marker was detected even though the product formation was not initiated by adding IPTG. For the reference L-phenylalanine production process in the STR, this signal was prior induction stable around 842.03 ± 71.25 and increased in the subsequent product formation phase by approximately 70% within the first 14 h. This trend agrees with the product formation rate’s development, reaching its maximum of 35.94 mg g_CDW_^−1^ h^−1^. Afterward, a decline of both, the mCardinal2 fluorescence, and the specific product formation rate was observed. For both TCB cultivations, the increase in median fluorescence intensity of mCardinal2 was more pronounced than in the STR process. When the mean residence time within the CFI was set to 102 s, fluorescence increased by 107% 16 h post-induction, and for the cultivation where the residence time was extended to 403 s inside the CFI, median fluorescence intensity increased even by 125% also 16 h after IPTG addition. However, product formation rates did not perfectly align with the trend of mCardinal2 fluorescence, reaching their maximum 26.8 h post-induction with 30.95 mg g_CDW_^−1^ h^−1^, or 30 h post-induction with 43.48 mg g_CDW_^−1^ h^−1^. Furthermore, it should be mentioned that fluorescence values maintain high intensities until the end of the cultivations, although product formation rates decline.

### 3.3. Single-Cell Fluorescence Distributions During L-phenylalanine Production in STR and TCB

**Figure A2:**
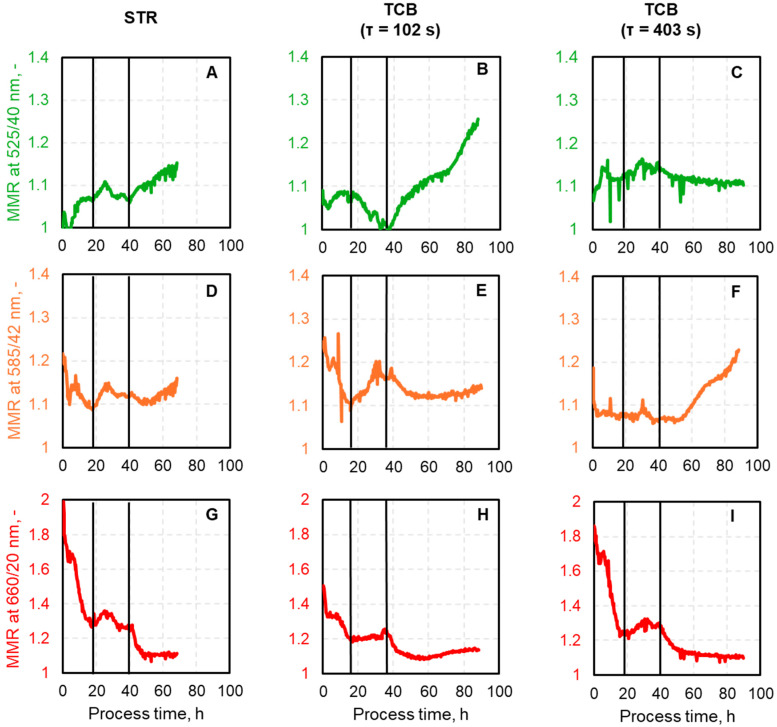
Real-time mean-to-median ratios (MMR) during the L-phenylalanine production process with *E. coli* FUS4 (pF81_Kan_) 3RP in an STR (first column), a TCB with τ = 102 s in the CFI (second column), and a TCB with τ = 403 s in the CFI (third column). (**A**–**C**) show the MMR of the green fluorescing mEmerald. (**D**–**F**) illustrates the ratios of the orange fluorescing CyOFP1. The graphs (**G**–**I**) depict the MMR of the red fluorescing mCardinal2. The first vertical line indicates the end of the batch and the start of the biomass production phase, while the second vertical line marks the start of the product formation phase.

Besides the median fluorescence intensities, which give a fair approximation of how the whole population responds to environmental changes, the single-cell analysis gives information about the response of individual cells, which is useful with respect to the investigation of phenotypic population heterogeneity. Hence, histogram distribution plots of the fluorescence intensity of mEmerald (growth), CyOFP1 (oxygen availability), and mCardinal2 (product formation) were generated. Additionally, mean-to-median ratios (MMR) will be used to support interpretation, where higher MMR indicates right skewed distributions and vice versa (Additional Figure A2).

**Figure 5:**
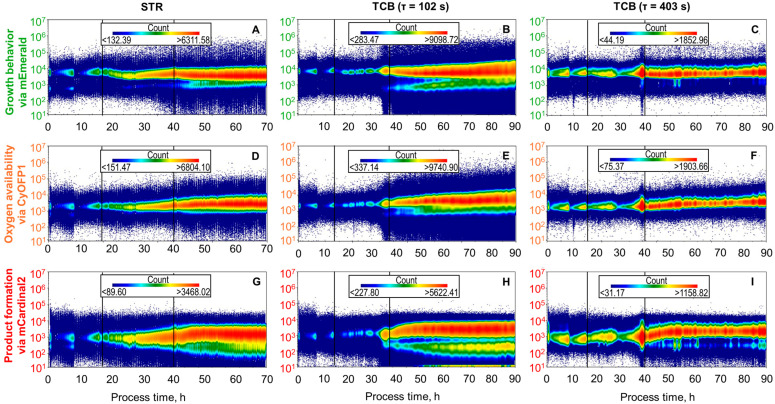
Histogram distributions from automated real-time flow cytometry where fluorescence intensity is plotted against the process time during the L-phenylalanine production process with *E. coli* FUS4 (pF81_Kan_) 3RP in an STR (first column), a TCB with τ = 102 s in the CFI (second column), and a TCB with τ = 403 s in the CFI (third column). Histogram distributions of *rrnB*-mEmerald are shown in the graphs (**A**–**C**), of *narGHIJ*-CyOFP1 in (**D**–**F**) and of *aroFBL*-mCardinal2 in (**G**–**I**). The first vertical line indicates the end of the initial batch phase, while the second vertical line the beginning of the L-phenylalanine production phase. Each plot includes a fluorescence count legend indicating population density.

**Single-cell response for expression of the growth behavior marker.** The growth behavior was monitored by the green fluorescing mEmerald detected at 525/40 nm (Figure 5A–C). The fed-batch process in the STR revealed during the initial batch phase bimodal distributions, in which the main *E. coli* population exhibited fluorescence intensities ranging from 2 ∙ 10^3^ to 4 ∙ 10^4^ and the smaller subpopulation fluorescences between 1 ∙ 10^2^ and 2 ∙ 10^3^. It can furthermore be noted that the fraction of the adjacent subpopulation decreased as batch cultivation proceeded, starting at around 26% and having only 7% at the end of the batch phase. The average MMR during this phase was 1.03 ± 0.03. During the first biomass production phase, the main population shifts to lower fluorescence intensities ranging from 2 ∙ 10^3^ to 3 ∙ 10^4^ and concluding at a range between 2 ∙ 10^3^ to 1 ∙ 10^4^. The subpopulation maintained exhibiting the same fluorescence intensity and the percentual amount. During the second phase of biomass production, fluorescence intensity distributions remained steady, with the only difference that the subpopulation shrinks and only around 4% of the population exhibited lower fluorescence intensities. With the induction of L-phenylalanine production, the main population gets wider in addition to the fusion of the subpopulation with the main population at a bioprocess time of around 50 h, leading to a tailing towards lower fluorescence intensities with MMR of 1.11 ± 0.02.

When mixing insufficiencies were simulated in the TCB, monomodal and very uniform distributions were observed at the lower mean residence time in the CFI, exhibiting fluorescence intensities between 2 ∙ 10^3^ and 3 ∙ 10^4^ and having an MMR of 1.07 ± 0.01 during the initial batch phase. With the start of the biomass production phase, tailing towards lower fluorescence intensities became obvious, which make up around 2% of the total population. However, as the second phase of biomass production proceeded, a bimodal distribution was formed over the process time, having around 12% of the total population at the end of the biomass production phase exhibiting intensities between 1 ∙ 10^2^ and 2 ∙ 10^3^. The MMR stayed constant at 1.05 ± 0.03 during the biomass production phase. With the induction of L-phenylalanine production, the subpopulation became proportionally larger compromising around 15% of the total population. As L-phenylalanine production proceeds, the smaller fraction of cells merged with the main population forming one population with extended tailing towards lower fluorescence intensities, also provable with rising MMR which reached at the end of the cultivation the maximum of 1.26.

In comparison, the extension of the mean residence time inside the CFI led during the batch phase to a monomodal and very uniform distribution, exhibiting fluorescence intensities between 2 ∙ 10^3^ and 3 ∙ 10^4^. During both biomass production phases, the distribution was very narrow showing intensities between 2 ∙ 10^3^ and 3 ∙ 10^4^, whereby around 2% of the population exhibit less fluorescence which can be seen as tailing. This observation is supported by MMR values of 1.11 ± 0.02 and 1.14 ± 0.01, respectively. With the start of the product formation phase, the distribution remained unchanged and with MMR values of 1.11 ± 0.01.

**Single-cell response for expression of the oxygen availability marker.** The oxygen availability of the *E. coli* cells was monitored by the orange fluorescing CyOFP1 detected at 585/42 nm (Figure 5D–F). The first eight hours of the initial batch phase in the STR showed a formation of a subpopulation at lower fluorescence intensities ranging from 1 ∙ 10^2^ to 1 ∙ 10^3^. First, this subpopulation made up 25% of the whole population but decreased as the batch phase concluded and diminished at the end of this phase. During the biomass production phase as well as the product formation phase, the distributions maintained monomodal and showed fluorescence intensities between 2 ∙ 10^2^ and 2 ∙ 10^4^, and MMR of 1.12 since the beginning.

During the fed-batch cultivation in the TCB with a mean residence time of 102 s in the CFI, the population exhibited fluorescence intensities between 8 ∙ 10^2^ and 2 ∙ 10^4^ during batch phase and biomass production phase and showed even distribution with MMR of 1.08 ± 0.01. With the onset of the second phase of biomass production, the distribution transformed gradually to a bimodal distribution, where at the end of this process phase around 10% of the *E. coli* cells showed intensities between 1 ∙ 10^2^ to 1 ∙ 10^3^. With the beginning of the L-phenylalanine production phase, the bimodal distribution slowly transforms into a broad left skewed monomodal distribution having fluorescence intensities between 2 ∙ 10^2^ to 2 ∙ 10^4^. Furthermore, MMR increases steadily from 1.07 and reaches a value of 1.23 at the end of the L-phenylalanine production process.

CyOFP1 expression during the TCB cultivation with a mean residence time of 403 s in the CFI showed narrow distributions during batch and biomass production phase, expressing intensities between 2 ∙ 10^2^ and 2 ∙ 10^4^. It can be noted that the distributions broadened immense within the last five hours of biomass production, exhibiting fluorescence intensities ranging from 7 ∙ 10^2^ to 10^4^. Upon induction with 0.3 mM IPTG, distributions constricted and shifted towards higher fluorescence intensities, expressing fluorescence intensities between 1 ∙ 10^3^ and 1 ∙ 10^4^. Throughout the fed-batch cultivation, MMR remains constant at a value of 1.14 ± 0.03.

**Single-cell response for expression of the product formation marker.** Regarding the product formation monitored via mCardinal2, detected at 660/20 nm, the fluorescence intensity distribution was the most left skewed, starting at an MMR of 1.99 and reaching at the end of the batch phase an MMR of 1.27 (Figure 5G–I). In the first eight hours of the cultivation process, bimodal distributions were detected. During both biomass production phases, the distributions became continuously monomodal, exhibiting fluorescence intensities between 3 ∙ 10^1^ and 2 ∙ 10^4^ and showing less tailing, noticeable through the constant MMR with 1.30 ± 0.03. Upon induction with 0.3 mM IPTG for L-phenylalanine formation, an immediate shift of the distributions towards higher intensities was observed. Thereby, approximately 93% of the population exhibited intensities ranging from 5 ∙ 10^2^ to 2 ∙ 10^4^, while around 7% were tailing towards lower fluorescences ranging from 2 ∙ 10^1^ to 5 ∙ 10^2^. Regarding the MMR a drop from 1.26 to 1.11 within the first nine hours of product formation was obvious. Afterwards, MMR remains steady at 1.11 ± 0.01.

When simulating mixing insufficiencies in the TCB with a mean residence time of 102 s in the CFI, distributions were monomodal during batch phase exhibiting fluorescence intensities between 8 ∙ 10^1^ and 2 ∙ 10^4^. Similar to the cultivation in the STR, MMR decreased steadily during this phase, starting at 1.51 and ending at 1.21. With the start of biomass production phase two, the fluorescence intensity distributions gradually formed a shoulder at intensities ranging from 2 ∙ 10^1^ to 2 ∙ 10^2^ making up to 15% of the total population. MMR values remained steady at around 1.21 ± 0.02. The induction with IPTG for L-phenylalanine synthesis led immediately to a shift to higher fluorescence intensities and the formation of a subpopulation. Herby around 80% of the population exhibited intensities ranging from 5 ∙ 10^2^ and 2 ∙ 10^4^ and 20% fluorescence intensities between 1 ∙ 10^1^ and 4 ∙ 10^2^. Starting at a process time of around 50 h, a third subpopulation arose compromising 5% and having fluorescence intensities ranging from 0 to 1 ∙ 10^1^. As L-phenylalanine production proceeded, the fraction of cells with very low fluorescence increased with 7% at process termination. The MMR values showed a steady during approximately the first five hours of L-phenylalanine formation from 1.24 to 1.13. Afterwards, MMRs stay relatively constant at 1.11 ± 0.02 until process termination.

Extension of the mean residence time inside the CFI to 403 s regarding the mCardinal2 expression, bimodal distributions were detected during the first eight hours of the batch phase with simultaneous shift to lower intensities. Afterwards, distributions remained steady at intensities between 2 ∙ 10^2^ and 1 ∙ 10^4^. MMRs decreased almost linearly from 1.84 to 1.23 during this phase. During the biomass production phase, the distributions remained monomodal but were slightly right-skewed, as seen by the average MMR of 1.28 ± 0.03. With start of the product formation phase, a distinct subpopulation arose, while the main population with around 95% shift within nine hours to higher fluorescence intensities ranging from 7 ∙10^2^ to 2 ∙ 10^4^ in conjunction with a 10% decline in MMR. The subpopulation, which accounts for approximately 5% of the total population, exhibits intensities between 6 ∙ 10^1^ and 7 ∙ 10^2^ and remains unchanged until the end of the L-phenylalanine production process.

### 3.4. Impact of Residence Time Versus Exposure Frequency on Population Heterogeneity

**Figure A3:**
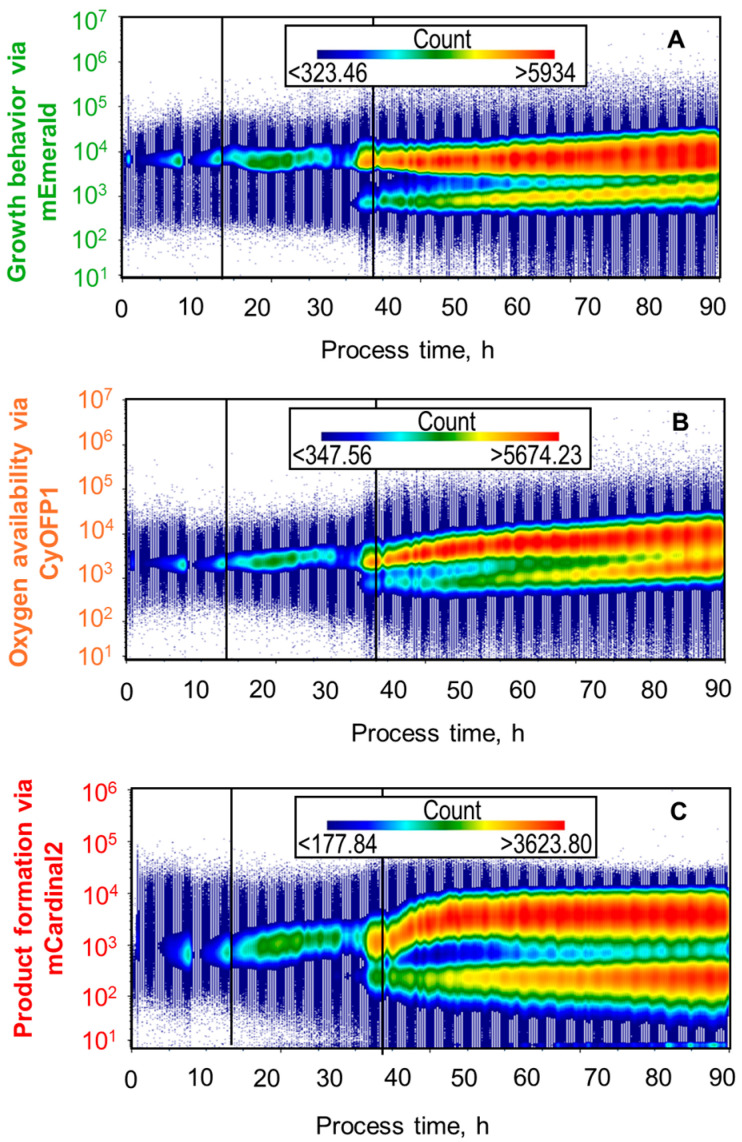
Histogram distributions from ART-FCM showing fluorescence intensity distributions over process time during the L-phenylalanine production process with *E. coli* FUS4 (pF81_Kan_) 3RP in an TCB with τ = 102 s in the CFI. The total initial volume was increased from 1.35 L (0.9 L STR + 0.45 L CFI) to 2 L (1.55 L STR + 0.45 L CFI). This volume increase reduced the frequency of exposure to the CFI from 4.1 to 2.8 cycles within the 20 min ART-FCM sampling interval. Histogram distributions of *rrnB*-mEmerald are shown in graph (**A)**, (**B**) shows the distribution of *narGHIJ*-CyOFP1 and (**C**) shows the fluorescence distribution of *aroFBL*-mCardinal2. The first vertical line indicates the end of the initial batch phase, while the second vertical line the beginning of the L-phenylalanine production phase. Each plot includes a fluorescence count legend indicating population density.

The aim of this study was to investigate how the residence time in the non-controlled compartment within the CFI influences microbial population heterogeneity. Using ART-FCM, a counterintuitive trend was observed: the longer residence time (403 s) in the CFI resulted in a more coordinated population response, noticeable through a narrower fluorescence distribution compared to the shorter residence time (102 s). To further investigate whether this observation is due to the extended exposure time or to the frequency of exposure to the confinement zone in the CFI, an additional experiment was conducted. For that, the total volume of the bioreactor was increased from 1.35 L (0.9 L STR + 0.45 L CFI) to 2.0 L (1.55 L STR + 0.45 L CFI), while maintaining a residence time of 102 s in the CFI. This adjustment reduced the frequence of exposure to the limiting conditions inside the CFI from approximately 4.1 to 2.8 times during the 20 min ART-FCM interval. Despite this change in exchange frequency, the fluorescence distribution profiles remained largely unchanged compared to those obtained with the smaller total volume at the same residence time (Figure A3).

## 4. Discussion

### 4.1. Advantages of Automated Real-Time Flow Cytometry

The implementation of automated real-time flow cytometry (ART-FCM) in this study provided an unprecedented temporal resolution for monitoring cellular responses during L-phenylalanine production with *E. coli*. With the integration of the OC-300 automation add-on, autonomous, high-frequency sampling every 20 min enabled the capture of transient and dynamic physiological events that might have been missed with conventional at-line flow cytometry [14,16]. This highlights the significant enhancement compared to the work of Hoang et al. [10], where at-line flow cytometry was used to monitor phenotypic population heterogeneity during the L-phenylalanine process. Beyond enhanced monitoring, the high-resolution fluorescence data also opens opportunities for development of dynamic, feedback-based process control strategies. By capturing real-time population dynamics, ART-FCM creates the necessary conditions for adaptive process adjustments based on current cellular responses.

### 4.2. Comparison of Process Performance

Scale-down bioreactors are valuable tools to mimic spatial heterogeneities encountered in large-scale fermentation processes [4,7]. In this study, a two-compartment bioreactor, consisting of a stirred-tank bioreactor and a coiled flow inverter, was applied, testing mean residence times of 102 s and 403 s in the CFI [10]. In this work, the cultivation was first conducted in a well-mixed 3.6 L STR with an initial working volume of 1.35 L and later transferred to a TCB with the same initial working volume but with a ratio of 2:1 between STR and CFI. During the initial batch phase, all cultivations displayed comparable growth behavior, reaching biomass concentrations between 1.5–1.7 g L^−1^. This suggests that early growth was not strongly affected by the scale-down configuration or mean residence time in the CFI, as the bioreactors remained largely homogeneous prior to nutrient limitation. Differences began to emerge during the first biomass production phase under exponential feeding. The STR achieved the highest biomass concentration (5.0 g L^−1^), while the TCBs with τ = 102 s and τ = 403 s reached slightly lower values (4.6 g L^−1^, and 4.1 g L^−1^, respectively). This trend continued into the second biomass production phase, where the STR reached over 21 g L^−1^ biomass, whereas the TCB cultivations lagged behind, reaching 15.9 g L^−1^ (102 s) and 14.1 g L^−1^ (403 s). After IPTG induction, L-phenylalanine production began with different kinetics across the bioreactors. In the STR, product formation was initiated within six hours post-induction and reached the maximum concentration of 21 g L^−1^ within 27 h. In contrast, both TCB setups exhibited delayed production onset, starting 12 h after induction. Moreover, while the maximum L-phenylalanine concentrations in the TCBs were comparable (20 g L^−1^), they were achieved significantly later, around a process time of 90 h. Despite the delayed onset, the final product concentrations were only marginally lower in the TCB cultivations compared to the STR. In the STR, both OUR and CER followed a classical pattern, peaking during biomass production and increasing only slightly during product formation. In contrast, in the TCBs, particularly at the longer mean residence time in the CFI, OUR peaked later, suggesting that respiration remained elevated, possibly due to stress-induced overflow metabolism or compensatory responses to oxygen fluctuations. This aligns with previous observations in scale-down bioreactors, where fluctuating oxygen conditions resulted in inefficient metabolism and increased maintenance energy demand [29]. The overall trends observed in this study align well with those reported by Hoang et al. [10], who utilized an identical STR-CFI setup with a residence time of 102 s. In their STR cultivation, a notably higher biomass concentration of 33.8 g L^−1^ was achieved, while the maximum L-phenylalanine concentration was comparable at 21.5 g L^−1^. Notably, the TCB cultivation (τ = 102 s) showed a 21% reduction in biomass compared to the STR, indicating a more pronounced impact of spatial heterogeneities on cell growth than observed in the present study, where no reduction in biomass was observed.

### 4.3. Comparison of the Median Fluorescence Intensities

The application of ART-FCM enabled continuous tracking of median fluorescence intensities of the reporter proteins mEmerald (growth), CyOFP1 (oxygen availability), and mCardinal2 (product formation). Comparing fluorescence trends across the STR and TCB setups with different residence times allowed for detailed insights into how environmental fluctuations impact cellular physiology over time.

**Monitoring growth behavior with green fluorescing mEmerald.** The green fluorescence reporter mEmerald, coupled to the *rrnB* operon, reflects ribosomal activity and, thus, growth-related transcription. As expected, all cultivations showed maximum mEmerald fluorescence signals at the end of the batch phase, coinciding with the highest specific growth rate. During the first biomass production phase a decline in median fluorescence intensities was observed, again, correlating to the specific growth rate. However, after changing to the energy-richer feed medium, which contained 400 g L^−1^ glycerol, an increase in fluorescence was observed in all three fed-batch processes, although the growth rate remained set at 0.1 h^−1^. The study by Murray et al. [30] showed that the *rrnB* P2 promotor, which is part of the *rrnB* operon, is not only regulated by growth rate but also amino acid availability. This suggested that the increase in mEmerald expression might be due to the increased availability of the substrate glycerol and the essential amino acids L-phenylalanine and L-tyrosine [30]. During the product formation phase in the STR, median intensities of mEmerald gradually declined, in line with near-zero growth rates as cells shifted their metabolic activity towards L-phenylalanine production. However, in both TCB cultivations a re-increase in mEmerald became obvious after around 30 h of product formation, despite negligible growth. One possible explanation is the accumulation of the stable mEmerald protein in the absence of dilution by cell division. This is supported by the findings from Andersen et al. [31], who investigated the in vivo stability of a related GFP variant (GFPmut3*) in *E. coli* and reported that the mature protein remained fluorescent for several weeks. Based on these observations, they conservatively estimated the in vivo half-life of GFPmut3* to be greater than 24 h, highlighting the strong stability of GFP-derived proteins such as mEmerald [31].

**Monitoring oxygen availability with orange fluorescing CyOFP1.** The CyOFP1 fluorescence reporter monitors oxygen availability through the expression of the *narGHIJ* operon. Given that the DO levels in the STR were tightly controlled above 30% air saturation, CyOFP1 fluorescence remained relatively stable during the batch and early biomass production phases. However, during the end of biomass production phase two, DO could not be held above 30%. Here, an increase in CyOPF1 expression could be observed. This increase in CyOFP1 intensity is consistent with the known response of *narGHIJ* operon to low oxygen conditions, as the operon is upregulated in response to respiratory stress, enabling cells to adapt to suboptimal oxygen levels [32]. Except that, cultivation in the STR did not show major changes in fluorescence, confirming oxygen availability remained sufficient throughout the whole fed-batch production process.

In contrast, the two-compartment bioreactor cultivations revealed substantially different CyOFP1 expression dynamics. Although the DO probe was placed in the aerated STR, the observed increases in CyOFP1 fluorescence in both TCB conditions suggest significant localized oxygen limitation within the non-aerated CFI. During the early phases of cultivation, fluorescence remained relatively low. However, as L-phenylalanine production started, a notable rise in CyOFP1 fluorescence occurred at both mean residence times in the CFI. This rise in fluorescence cannot be attributed to changes in the DO levels in the STR, which remained strictly controlled, but instead likely reflects oxygen depletion within the CFI. It can be assumed that this increase is likely due to the increase in cell dry weight over time. As biomass accumulates during the later phases of the fed-batch process, the total oxygen demand of the *E. coli* culture increases. This effect has been reported in previous scale-down studies, where local oxygen limitation becomes more severe at high biomass concentrations [33]. Notably, after approximately 70 h of cultivation, CyOFP1 fluorescence showed first stagnation and then a secondary increase in both TCB setups. This may indicate that cells, while no longer growing substantially, continue to exhibit high respiratory activity due to product synthesis and maintenance metabolism, further exacerbating local oxygen demand. Elevated oxygen uptake rates measured during the product formation phase support this interpretation.

**Monitoring of product formation with red fluorescing mCardinal2.** The expression of the red fluorescing mCardinal2, used as a reporter for L-phenylalanine production, is together with the gene cluster *aroFBL* under the control of the IPTG-inducible P_tac_ promotor and thus, produced when cells are induced with IPTG. In the STR, the induction with IPTG triggered a rapid increase in mCardinal2 fluorescence. The fluorescence reached the maximum after approximately 10 h, coinciding with a peak in the specific product formation rate. For the L-phenylalanine production process in the STR, it can be concluded that the temporal overlap between rising fluorescence and product formation rate suggests that mCardinal2 is a reliable proxy for production performance, when promoter induction is not hindered by environmental gradients.

One of the most intriguing observations in this study was the early increase in mCardinal2 fluorescence following IPTG induction in all cultivations. However, in the TCB setups, this was contrasted by a pronounced delay in L-phenylalanine production. In the STR, L-phenylalanine synthesis started within six hours post-induction, closely following mCardinal2 expression. In contrast, TCB cultivations showed a decoupling of fluorescence signal and product formation. Given that mCardinal2 is located downstream of the *aroFBL* gene cluster on the same P_tac_-promotor, its expression should, in theory, correlate with the transcription of the upstream L-phenylalanine biosynthesis genes. However, this correlation was disrupted under fluctuating cultivation conditions. The fluorescent protein mCardinal2 is structurally simple, and does not rely on extensive precursor or cofactor availability [34]. In contrast, L-phenylalanine biosynthesis depends on multiple rate-limiting factors, including sufficient levels of precursors (phosphoenolpyruvate, erythrose-4-phosphate), ATP, and NADPH, as well as enzymatic activity of the AroF, AroB, and AroL proteins [35]. The delayed onset of L-phenylalanine production thus likely reflects a metabolic inefficiency under dynamic conditions. This observation aligns with the findings of Bafna-Rührer et al. [29], showing that oscillating levels of glucose and oxygen significantly alter both gene expression and physiological behavior in *E. coli*. Therefore, cells may fully express mCardinal2 while being metabolically unable to synthesize L-phenylalanine under suboptimal conditions.

### 4.4. Investigation of Population Heterogeneity Under Fluctuating Conditions

The implementation of ART-FCM in this study enabled high-resolution single-cell analysis, revealing subtle and dynamic heterogeneity in different cultivation setups. While median fluorescence values offer valuable insight into population-level trends, the fluorescence intensity distributions of mEmerald, CyOFP1, and mCardinal2 provide a deeper understanding of phenotypic diversification at the single-cell level.

**Monitoring growth behavior with green fluorescing mEmerald.** The green fluorescent protein mEmerald, linked to the *rrnB* operon, served as an indicator of growth activity and ribosomal transcription. In the STR, the population exhibited initial bimodality during the batch phase, with a subpopulation at low fluorescence levels. This bimodality gradually resolved into a monomodal distribution by the end of the batch phase, indicating synchronization of cellular activity under homogeneous conditions. Minor tailing reappeared during the product formation phase, reflecting reduced but uniform growth.

In contrast, under the short mean residence time condition in the CFI of the TCB, bimodal distributions were observed for mEmerald during biomass production. This suggests that environmental fluctuations caused by the rapid cycles between the well-mixed STR and the poorly mixed CFI led to asynchronous growth patterns across the population. A subpopulation of cells, likely experiencing temporary nutrient or oxygen limitation in the CFI, exhibited low mEmerald fluorescence, while the majority maintained normal growth activity. This increase in heterogeneity was reflected by a higher MMR which peaked during the product formation phase when growth slowed dramatically.

Notably, when the mean residence time was increased to 403 s in the CFI, mEmerald distributions remained remarkably uniform throughout. Although minor left-skewed tailing appeared, no distinct subpopulations formed. MMR values rose slightly, but without sharp transitions. This suggests that longer exposure to CFI conditions allowed cells to transcriptionally adapt in a coordinated manner, resulting in a homogenized expression state, possibly by invoking the RpoS-mediated general stress response pathway, which reprograms the cell toward maintenance and robustness rather than rapid growth [36,37].

**Monitoring oxygen availability with orange fluorescing CyOFP1.** The CyOFP1 fluorescence, which reports on oxygen-limitation-driven transcription via the *narGHIJ* promoter, followed expected trends. In the STR, distributions remained narrow throughout, confirming consistent oxygen availability. In contrast, the TCB setups showed increasing CyOFP1 expression during biomass production, with higher variability in the 102 s bypass TCB. This again points to the impact of short-term environmental fluctuations, which differentially activated the oxygen stress response across the population. However, the 403 s bypass TCB cultivation showed more symmetrical and monomodal CyOFP1 profiles, suggesting that the population underwent a coordinated adaptation to sustained oxygen limitation. These results support the idea that predictable environmental stress elicits synchronized transcriptional responses, rather than rapid cycling between contrasting environments, which increases heterogeneity.

**Monitoring of product formation with red fluorescing mCardinal2.** Following induction during STR cultivation, the fluorescence distributions were symmetrical and shifted uniformly toward higher intensities. Over time, the population remained largely unimodal with minor tailing toward lower fluorescence, suggesting a mostly homogeneous transcriptional response to IPTG induction under well-controlled environmental conditions. In contrast, the two-compartment bioreactor (TCB) operated at a 102 s mean residence time in the CFI revealed pronounced subpopulation dynamics post-induction. The histogram distributions of mCardinal2 became increasingly complex, with up to three distinguishable subpopulations by the mid-to-late stages of cultivation: a fraction of cells with high fluorescence signals, probably fully induced cells, a subpopulation with lower fluorescence signals, probably partially induced cells, and a low- or non-fluorescent fraction, which are potentially cells that are unable to respond to induction. This phenotypic diversification suggests that rapid cycling between the well-controlled STR and the uncontrolled zone in the CFI apparently leads to asynchronous responses to the induction with IPTG. In contrast, the TCB at 403 s mean residence time in the CFI presented a more stable but still bimodal fluorescence distribution following IPTG induction. While the main population shifted uniformly toward high mCardinal2 expression, a persistent low-fluorescence subpopulation (around 5%) remained throughout the product formation phase. Notably, unlike in the 102 s bypass condition, this low-expressing group did not expand or fragment into additional subgroups.

### 4.5. Influence of Residence Time on Population Heterogeneity

The findings from this study provide evidence that the residence time within the CFI is the key source of enhanced population heterogeneity. The additional volume adjustment experiment demonstrated that reducing the frequency of exposure, while keeping the residence time constant, did not substantially alter the fluorescence distributions. This observation suggests that the residence time itself, rather than the frequency of cellular exposure to the CFI, plays a more critical role. The extended residence time in the CFI likely permits a more unified adaptation across the population, resulting in more synchronized behavior. Conversely, shorter residence times may cause frequent and abrupt shifts between controlled and uncontrolled conditions, which could foster more diverse individual responses and thus higher population heterogeneity.

## 5. Conclusions

This study demonstrates the power of ART-FCM for high-resolution monitoring of microbial population dynamics in bioprocesses. By enabling autonomous, high-frequency sampling during the cultivation of L-phenylalanine-producing *E. coli* in scale-down bioreactors, we obtained temporally resolved insights into adaptation mechanisms at the single-cell level. Fluorescence reporters for monitoring growth (*rrnB*-mEmerald), oxygen availability (*narGHIJ*-CyOFP1), and product formation (*aroFBL*-mCardinal2) allowed detailed tracking of phenotypic changes in response to environmental fluctuations in the two-compartment bioreactor. One of the most important findings of this study was the influence of residence time in the uncontrolled CFI on the cells. Apparently, longer residence time promotes more uniform cellular responses, suggesting coordinated stress adaptation. It is not yet fully understood whether population heterogeneity is detrimental to bioprocesses on an industrial scale. These results emphasize the need for well-designed scale-down studies to uncover and understand such effects. Ultimately, integrating real-time single-cell monitoring into bioprocess development can help anticipate and manage cellular responses, improving scalability and robustness in industrial fermentation processes.

## Data Availability

The original contributions presented in this study are included in the article. Further inquiries can be directed to the corresponding author.

## Abbreviations

The following abbreviations are used in this manuscript:

STR	Stirred-tank bioreactor
CFI	Coiled flow inverter
ART-FCM	Automated real-time flow cytometry
TCB	Two-compartment bioreactor
DO	Dissolved oxygen
SDB	Scale-down bioreactor
OD_600_	Optical density at 600 nm
CDW	Cell dry weight
PBS	Phosphate-buffered saline solution
HPLC	High-performance liquid chromatography
MMR	Mean-to-median ratio
